# Genomic Aberrations in an African American Colorectal Cancer Cohort Reveals a MSI-Specific Profile and Chromosome X Amplification in Male Patients

**DOI:** 10.1371/journal.pone.0040392

**Published:** 2012-08-06

**Authors:** Hassan Brim, Edward Lee, Mones S. Abu-Asab, Mohamed Chaouchi, Hadi Razjouyan, Hassanzadeh Namin, Ajay Goel, Alejandro A. Schäffer, Hassan Ashktorab

**Affiliations:** 1 Department of Medicine and Cancer Center, College of Medicine, Department of Pathology, Howard University, Washington, D.C., United States of America; 2 National Cancer Institute, National Institutes of Health (NIH), Department of Health and Human Services (DHHS), Bethesda, Maryland, United States of America; 3 Department of Physiology and Biophysics, Georgetown University School of Medicine, Washington, D.C., United States of America; 4 Baylor Research Institute and Sammons Cancer Center, Baylor University Medical Center, Dallas, Texas, United States of America; 5 National Center for Biotechnology Information, National Institutes of Health (NIH), Department of Health and Human Services (DHHS), Bethesda, Maryland, United States of America; University Hospital Carl Gustav Carus, Germany

## Abstract

**Objective:**

DNA aberrations that cause colorectal cancer (CRC) occur in multiple steps that involve microsatellite instability (MSI) and chromosomal instability (CIN). Herein, we studied CRCs from AA patients for their CIN and MSI status.

**Experimental Design:**

Array CGH was performed on 30 AA colon tumors. The MSI status was established. The CGH data from AA were compared to published lists of 41 TSG and oncogenes in Caucasians and 68 cancer genes, proposed via systematic sequencing for somatic mutations in colon and breast tumors. The patient-by-patient CGH profiles were organized into a maximum parsimony cladogram to give insights into the tumors' aberrations lineage.

**Results:**

The CGH analysis revealed that CIN was independent of age, gender, stage or location. However, both the number and nature of aberrations seem to depend on the MSI status. MSI-H tumors clustered together in the cladogram. The chromosomes with the highest rates of CGH aberrations were 3, 5, 7, 8, 20 and X. Chromosome X was primarily amplified in male patients. A comparison with Caucasians revealed an overall similar aberration profile with few exceptions for the following genes; THRB, RAF1, LPL, DCC, XIST, PCNT, STS and genes on the 20q12-q13 cytoband. Among the 68 CAN genes, all showed some level of alteration in our cohort.

**Conclusion:**

Chromosome X amplification in male patients with CRC merits follow-up. The observed CIN may play a distinctive role in CRC in AAs. The clustering of MSI-H tumors in global CGH data analysis suggests that chromosomal aberrations are not random.

## Introduction

Numerous studies have investigated the mechanisms of DNA changes leading to colorectal cancer (CRC), which is the third most common cancer in the US [1]. CRC incidence is high in African-Americans (AAs), among whom it causes a higher proportion of deaths than in other populations (1). Most CRC arise from adenomas, in a process described as adenoma-carcinoma sequence [2]. The initiation and progression of CRC is associated with alterations in the function of oncogenes and tumor suppressor genes.

Three major mechanisms of genomic instability in CRC have been described: microsatellite instability (MSI), chromosomal instability (CIN), and more recently CpG island methylation phenotype (CIMP). Excessive promoter methylation of hundreds of genes results in the CIMP is part of the epigenetic instability in CRC. More than one mechanism may occur in the same tumor. In MSI, which occurs in about 15% of CRC, DNA mismatch repair genes are either mutated or methylated leading to tumors with a microsatellite instability phenotype (denoted MSI-High, MSI-H, or MIN) [3].

In contrast, the CIN phenotype is characterized by global genomic rearrangements resulting from deletions, amplifications and translocations of chromosomal fragments [4]. CIN results from specific mutations or regulatory silencing of gene silencing and could manifest as structural defects involving centromeres or centrosomes, microtubule dysfunction, telomere erosion, chromosome breakage and failure of cell cycle checkpoints [5]. In this study, we focus on the two more studied mechanisms, MSI and CIN. The mechanism of MSI was first characterized in the context of a subcategory of CRC called hereditary non-polyposis colorectal cancer or Lynch syndrome, in which patients have heterozygous gremlin mutations of genes such as *MLH1* and *MSH3* [6]. The acquisition of recurrent chromosomal gains and losses during the progression from high-grade adenomas to invasive carcinomas has been repeatedly found in CIN CRC tumors [7]. CIN results from specific mutations or gene rearrangement and that could manifest as structural defects involving centromeres or centrosomes, microtubule dysfunction, telomere erosion, chromosome breakage and failure of cell cycle checkpoints (5). One of the earliest acquired genetic abnormalities during CRC progression involves chromosome 7 copy number gains which are observed in some colon adenomas as well [8]. At later stages of tumor progression, other specific chromosomal aberrations become more common, such as gains on chromosomes 8q, 20q [9], 7, 13 [10], [11] and copy number losses on chromosomes 8p, 17p, 18q [10], [12] 15q and 20q [13]. For some years, CIN and MSI tumors were considered as mutually exclusive, and it was thought that MSI tumors generally have stable, diploid karyotypes [14], [15]. However, recent studies have found that MSI and CIN can occur in the same tumor [16], [17]. Trautmann et al. found that at least 50% of MSI-H tumors have some degree of simultaneous chromosomal alterations [18]. Although evidence for some degree of CIN could be observed in the majority of MSI-H tumors, the pattern of specific gains and losses between MSI-H and MSS tumors is still poorly understood. MSI-H tumors tend to harbor gains of chromosomes 8, 12 and 13 and losses of 15q and 18q, while MSS tumors have a high degree and variable range of chromosomal aberrations [13], [18]. Chromosomal aberrations, like homozygous and heterozygous deletions or amplifications, alter the DNA copy number of large genomic regions or even whole chromosomal arms, leading to the inactivation of tumor suppressor genes or to the activation of oncogenes. Lassmann et al. studied 287 target sequences in Caucasian colorectal tumor cell DNA and found aberrations in specific regions of chromosomes 7, 8, 13, 17 and 20 [19].

**Table 1:** Clinico-pathological characteristics of the patients analyzed in this study.

Patient	Specimen#	Age	Sex	Stage	Location	Differentiation	MSI	#Aberrations
268	07-2378	94	F	1	R	Moderately	S	30
283	07-5430	54	M	1	R	well diff	S	35
308	09-1574	48	M	2	L	Moderately	S	10
270	07-3920	51	M	2	L	Moderately	S	99
269	07-3698	53	M	2	L	Moderately	S	65
267	07-1361	66	M	2	R	Moderately	H	63
272	07-4027	65	F	3	L	Moderately	S	30
277	07-5330	72	F	3	R	Moderately	S	31
275	07-4855	87	F	3	R	Moderately	S	26
285	08-2842	52	F	4	R	Moderately	S	13
287	08-3072	73	M	4	R	Moderately	S	8
2	05-3518	65	M	1	R	Moderately	S	2
1	05-3429	65	F	2	L	Moderately	S	13
5	05-4671	71	M	2	L	Moderately	S	29
14	06-4708	65	M	3	L	well diff	S	27
282	08-2321	55	F	3	R	Poorly	S	36
7	05-5288	73	F	3	R	Moderately	S	8
273	07-4527	71	M	3	R	Moderately	S	37
13	06-4383	53	M	3	R	Moderately	S	5
8	05-5581	61	M	3	R	Moderately	S	10
4	05-4211	57	M	3	R	Moderately	S	5
12	06-2689	53	F	4	R	Moderately	S	88
11	06-0477	51	F	2	L	Moderately	L	33
10	05-5770	69	F	2	L	Moderately	L	13
307	09-1637	53	F	3	R	Moderately	L	10
279	07-5443	60	M	3	R	Moderately	L	6
9	05-5659	54	F	2	R	Moderately	H	4
6	05-5026	83	F	3	R	Moderately	H	14
3	05-4203	68	F	3	R	Moderately	H	9
15	06-5215	64	M	3	R	Moderately	H	5

Studies that explore differentially expressed genes that cause tumorigenesis or tumor development may lead to discovering specific targets for cancer therapy and increase our understanding of the process of tumorigenesis. We have previously published results from a genome wide analysis of 15 AA CRC tumors [20] that microduplications are mainly present in chromosomes 20q, 8q, and 7q while microdeletions occur in 18q, 8p and Xp in AAs. The most frequently amplified region was 20q12-13 that includes the genes: *TNFSF6B, PTPN1, PRPF6* and *NCOA3*. The most frequently deleted genes were *LPL* (33%), *HIC1* (33%), and *BCL2* (27%) on chromosomes 8p22, 17p13.3 and 18q21.3, respectively. Our study indicated that there are recurrent aberrations in CRC involving chromosomes 20, 18, 17, 8, and 7 shared with Caucasian CRC patients. In addition, aberrations at chromosomes 11, 17p and X may be prominent in AAs.

Based upon these findings, we hypothesized that chromosomal aberrations in CRCs from AA patients, if validated in a larger cohort, could be useful for studying the racial differences and the disease disparity statistics in the AA population. Therefore, we investigated the CIN and status in a larger cohort of additional AA CRC patients and compared our results with the findings in Caucasians [19] as well as with a list of colon cancer genes established by Sjöblom et al. based on their sequencing of 13,023 genes in 11 colon tumors [21]. We also performed a parsimony phylogenetic analysis of all recorded genomic aberrations to identify genomic signatures that might associate with clinical and pathological characteristics of the analyzed CRCs. The general aim of this study was to identify the chromosomal aberrations in African-American CRCs to delineate the specific genomic events of CIN in this high risk population.

## Materials and Methods

### Ethics Statement

This study was approved by the Howard University Institutional Review Board, and written, informed consent was obtained from all participants.

### Patient selection

Fresh frozen archived samples were used. Colonic biopsies (n = 30) were obtained from African-American patients undergoing colonoscopy at Howard University Hospital. This study was approved by the Howard University Institutional Review Board. Clinical data collected on each patient included race, gender, associated past medical history, medication use, and family history of colorectal cancer. Patients were deemed eligible if colonoscopy resulted in a first diagnosis of colon cancer, confirmed by histopathology. From the medical records, clinical information was collected and recorded based on the American Joint Committee on Cancer (AJCC) staging system. All patients in this study were African Americans by self-report.

### Sample selection and DNA extraction for array comparative genomic hybridization (aCGH)

Fresh tumor blocks were cut into 5-µm thick sections on Superfrost slides (Fisher Scientific, Pittsburgh, PA). The tumor and normal areas were delineated by a pathologist using the matched hematoxylin and eosin (H&E) slide were microdissected from which DNA was extracted using Puregene kit according to the manufacturer's instructions (Qiagen, Germantown, MD). The goal of the microdissection was to minimize the cross-contamination of normal and tumor tissues, which could impact the outcome of the experiment.

### MSI analysis

DNA from the analyzed tumors was used as a template in PCR reactions with five primer pairs, corresponding to the standard panel for MSI detection in colon cancer samples (BAT25, BAT26, NR21, NR22 and NR27), as described previously [22], [23], [24]. Samples that showed at least two PCR fragments with sizes different from the wild type were labeled microsatellite instability high (MSI-H), those with only one instability marker were labeled microsatellite instability low (MSI-L) while those with all PCR fragments with the expected size were labeled as microsatellite stable (MSS) [22], [23], [24].

### Comparative genomic hybridization (CGH) experiments

In these experiments, we studied the chromosomal aberration profiles in the 30 CRC samples. Our reference control was either matched normal or commercially procured sex-matched normal DNA (Promega, Wisconsin, WI). Tissues were evaluated by a GI pathologist for analysis of histological features including the size, type, location and pathological criteria of the carcinomas. An oligo microarray-based chip containing 105,000 human probes (Agilent, Santa Clara, CA; www.agilent.com) was used for CGH analysis. For each aCGH experiment, 1.5 µg of reference DNA and 1.5 µg of tumor DNA were used. Briefly, the test and reference DNAs were digested with *Alu* I and *Rsa* I (Promega, Madison, WI), and purified with the QIAprep Spin Miniprep kit (QIAGEN, Germantown, MD). Test DNA (1.5 µg) and reference DNA (1.5 µg; Promega) were labeled by random priming with Cy5-dUTP and Cy3-dUTP, respectively, using the Agilent Genomic DNA Labeling Kit Plus. Following the labeling reaction, the individually labeled test and reference samples were concentrated using Microcon YM-30 filters (Millipore, Billerica, MA) and then combined. Following probe denaturation and pre-annealing with *Cot-1* DNA, hybridization was performed at 65°C with rotation for 40 hours at 20 rpm. Four steps were done with Agilent Oligo CGH washes: wash buffer 1 at room temperature for 5 min, wash buffer 2 at 37°C for 1 min, an acetonitrile rinse at room temperature for 1 min and a 30 sec wash at room temperature in Agilent's Stabilization and Drying Solution. All slides were scanned on an Agilent DNA microarray scanner. Data including Copy Number Variations were obtained by Agilent Feature Extraction software 9 and analyzed with Agilent Genomic Workbench 5.0 software, using the statistical algorithms z score and ADM-2 according to sensitivity threshold respectively at 2.5 and 6.0 and a moving average window of 0.2 Mb. Mapping data were analyzed on the human genome sequence using the NCBI database build 35 also known as hg17 (http://www.ncbi.nlm.nih.gov).

### Computational analysis of genes targeted by copy number aberrations

To determine whether specific genes were gained or lost in each tumor sample, we compared the genomic locations of those genes with the gained and lost intervals in the “IntervalBasedReport” produced (ADM-2) for each case by the array CGH software. To do this comparison, we developed UNIX scripts and programs in C and Perl. Part of the IntervalBasedReport is the magnitude of each gain or loss, which enabled us to filter the resultant results by order of their magnitude and keep only those events that were above the threshold of 1.2-fold for gains and below the threshold 0.8-fold for losses.

### Parsimony Phylogenetic Analysis of CGH Microarray Data

Microarray data analysis generally focused on specific genes of known relevance to the pathology at hand. Here, we have taken all chromosomal aberrations into consideration to conduct a parsimony phylogenetic analysis. Briefly, to find out the distribution of aberrations for each specimen in relation to the total aberrations of all specimens the following procedure was carried out: all aberrations of all the cancer specimens were summed up and the duplicates removed; each specimen's aberrations list was compared to the total list of aberrations and each aberration scored as present (1) or absent (0), this polarity assessment produced a new data matrix of CGH data. The new data matrix was processed for maximum parsimony with MIX algorithm (of the PHYLIP analytical package to produce the cladograms.

### Statistical analysis

Numerical data was expressed as mean ± standard deviation (SD). Student's t-test or one-way analysis of variance (ANOVA) were used for comparison of means. Categorical variables were compared using the chi-square test. P-values less than 0.05 were considered significant. Statistical analysis was performed using the SPSS 19.0 software package (IBM Corp., Somers, NY, USA).

**Table 2:** MSI analysis and association with clinical and demographical parameters.

	MSI			
	Stable (n = 21)	Low (n = 4)	High (n = 5)	P value
**Median age (25–75% interquartile)**	65 (53–71)	56.5 (51.5–66.7)	66.0 (59–75.5)	0.4
**Gender, no (%)**				0.4
Male	12(57.1)	1(25)	2 (40)	
Female	9 (42.9)	3 (75)	3 (60)	
**Location, no (%)**				0.2
Right	14(66.7)	2(50)	5 (100)	
Left	7 (33.3)	2 (50)	0	
**Stage**				0.7
One	3	0	0	
Two	5	2	2	
Three	10	2	3	
Four	3	0	0	

**Table 3:** Aberrations patterns in each chromosome in African American CRC tumors.

	Total aberrations	Amplif.	Del.	Amplif. Males	Amplif. Females	Del. Males	Del. Females
1	36	20	16	12	8	6	10
2	35	22	13	7	15	5	8
3	44	19	25	6	13	11	14
4	37	16	21	8	8	12	9
5	41	21	20	6	15	5	15
6	29	16	13	6	10	1	12
7	47	35	12	15	20	5	7
8	48	25	23	12	13	10	13
9	20	16	4	7	9	2	2
10	28	16	12	6	10	3	9
11	38	24	14	13	11	5	9
12	30	22	8	8	14	2	6
13	30	19	11	9	10	5	6
14	29	16	13	6	10	6	7
15	16	6	10	3	3	3	7
16	36	29	7	14	15	2	5
17	34	22	12	12	10	6	6
18	26	5	21	0	5	8	13
19	25	17	8	9	8	2	6
20	31	26	5	13	13	2	3
21	19	9	10	4	5	3	7
22	22	11	11	5	6	4	7
X	24	14	10	10	4	3	7
Y	9	2	7	2		7	

## Results

### Characteristics of the analyzed samples

Our study cohort was comprised of 30 colon cancers from AA patients. Males and females were equally represented in this group. The mean age was 63.5 [SD = 1.1]. The tumors were left-sided in 9 patients (5 males and 4 females) and right-sided in 21 patients (10 males and 11 females). Most tumors (n = 27) were moderately differentiated, one was poorly differentiated while two were well differentiated. Half the tumors (n = 15) were of stage 3, nine tumors were stage 2, three were stage 4, and three were stage 1 (Table 1).

### MSI analysis

MSI results were obtained for all patients in this study. Of these 30 samples, 21 were microsatellite stable, 4 were microsatellite instable-low (MSI-L) and 5 were MSI-H. All MSI-H tumors were proximal while the MSI-L tumors were equally distributed throughout the colon. No associations were found with other clinical and demographic data. (Table 2).

### Genomic alterations in various chromosomes

All chromosomes were harbored a spectrum of alterations in multiple tumors. The chromosomes that had the fewest aberrations were chromosomes 15 and 21, with 16 and 19 aberrations respectively; chromosome 8 had the most aberrations (n = 48). Other chromosomes with high aberration counts were chromosomes 3, 5 and 7 with 44, 41 and 47 aberrations respectively. Male and female patients showed a similar distribution of alterations except on three chromosomes: 1) chromosome X was primarily amplified in males (10/15 males vs. 4/15 females), 2) chromosome 20 was also primarily altered in male patients, and 3) chromosome 18 had more alterations in females.

### Genomic alterations per case

A total of 764 aberrations were reported for all samples (average of 25.46 per tumor). The tumor with the least number of aberrations had 2 while the one with most aberrations had 99. The patient with the highest number of aberrations was 51 year male with a stage 2 tumor, while the one with the least aberrations was a 65 year old male with a stage 1 neoplasm. Overall, the number of chromosomal aberrations did not appear to be either age- or stage–related (Tables 1 & 3). The 15 female patients had a total of 358 aberrations with an average of 23.8 per patient. Male patients had 406 aberrations with an average of 27 per tumor. The statistical analysis revealed that the number of aberrations per sample did not associate with any clinical or demographical parameters (Table 4). An exception to this rule was the MSI-H tumors that showed fewer aberrations when compared to non MSI-H CRC, but there were not enough MSI-H samples to achieve significance (Table 4).

### Comparison of the aCGH data with the CRC CAN genes

A comparison of the our aCGH data with a list of 68 genes identified through the sequencing of 11 colon cancer tumors revealed that all of these genes, except *ACTL9*, are altered in at least one of the 30 tumors analyzed here. The altered genes showed different frequencies and types of aberrations (Table 5). In comparison to Caucasians, the following genes were predominantly amplified in AA population: *ADAMST18, CD248, CSMD3, EPHB6, ERGIC3, EXOC4, GALNS, GNAS, KR73. LMO7, MLL3, MMP2, NF1RUNX1T1, SFRS6SLC29A1, SLC44A4TP53, UQCRC2*, and *ZNF442*. These genes were amplified in at least one third of the tested samples. Deletions were less prevalent and the most frequently deleted genes on the candidate list are: *ADAM29, APC, FBXW7, HAPLN1, NF1, SMAD2, SMAD4,* and *TP53*. *SMAD2* and *SMAD4* were deleted in 16 out of 30 samples (Table 5).

**Table 4:** Number of aberrations and associations with clinical and demographical data.

	Aberration Mean (SD)	P value
Gender		0.7
Male	27.0(28.7)	
Female	23.8 (20.6)	
Location		0.2
Right	21.1 (21.9)	
Left	35.4 (28.8)	
Stage		0.2
1	22.3(17.7)	
2	36.5(32.3)	
3	17.2(6–30)	
4	36.3 (44.8)	
MSI		0.3
Non-MSI-H	26.7(24.8)	
MSI-H	19.0 (24.9)	

**Table 5:** Comparison of AA data with those from Caucasian patients.

		Lassmann		African Americans (n = 30)	
Gene	Chromosome	Amplified (%)	Deleted (%)	Amplified (%)	Deleted (%)
*THRB*	3p24.3		32	13	3
*RAF1*	3p25		14	20	6
*RFC2*	7q11.2	36		60	3
*CYLN2*	7q11.23	36			
*MET*	7q31	23		60	0
*LPL*	8p22		23	10	40
*E2F5*	8q22-q21.3	36		53	0
*LPL*	8p22		23		
*EXT1*	8q24.11-q24.13	32		46	6
*MYC*	8q24.12-q24.13	36		46	0
*EGR2*	10q21.3		23	10	13
*DMBT1*	10q25.3-q26.1		23	3	16
*LRRC32*	11q13.5	32		10	13
*ATM*	11q22.3	27		10	16
*INS*	11p tel	32		26	6
*BRCA2*	13q12-q13	36		60	0
*RB1*	13q14	41		53	0
*MAP2K5*	15q23		32	3	20
*SP6*	17ptel		23	30	30
*TOP3A*	17p11.2			10	33
*LLGL1*	17p12-17p11.2		36	10	33
*FLII*	17p12-17p11.2		23	10	33
*HIC1*	17p13.3		32	20	30
*CTDP1*	18q tel		45	0	40
*LAMA3*	18q11.2		14	0	40
*BCL2*	18q21.3		23	0	40
*DCC*	18q21.3	32	18	0	50
*TPD52L2*	20qtel	27		76	3
*TOP1*	20q12-q13.1	32		56	0
*TNFRSF6B*	20q13	32		53	3
*NCOA3*	20q13	32		63	0
*AURKA*	20q13	36		56	0
*CSE1L*	20q13	27		63	0
*MYBL2*	20q13.1	32		63	0
*PTPN1*	20q13.1-q13.2	23		63	0
*CYP24A1*	20q13.2	36		63	0
*ZNF217*	20q13.2	32		63	0
*PRPF6*	20q13.3	27		60	3
*PCNT*	21qtel		18	16	20
*XIST*	Xq13.2	36		33	13
*STS*	Xp22.3		23	23	13
*KAL1*	Xp22.3	36		30	13

### Comparative analysis of aCGH data between AAs and Caucasians

**Figure 1:**
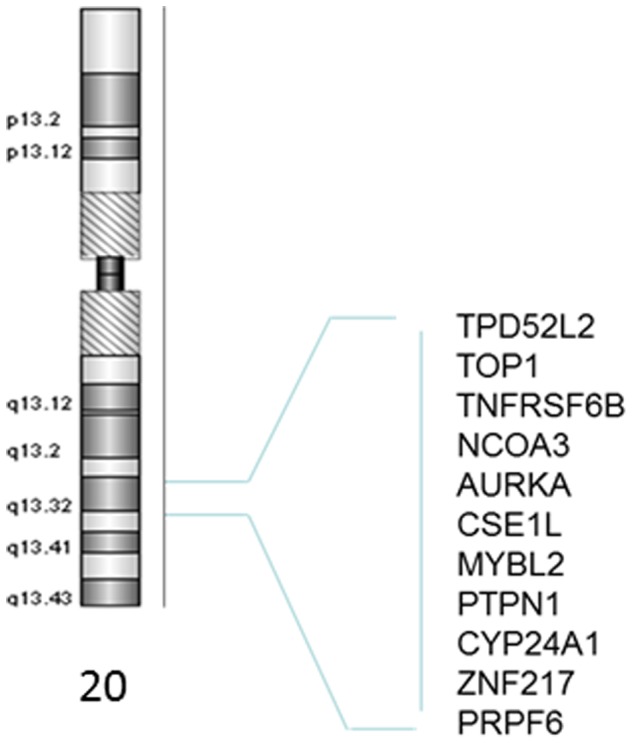
Schematic representation of the chromosome 20q13.0-13.3 and break apart amplified DNA includes genes located in this chromosomal region in sporadic African American colorectal cancer patients.

Lassmann et al. examined the aberration status of 41 known oncogenes and tumor suppressor genes in CGH data from 22 Caucasians [19]. We compared the outcome of their analysis with our data from African American patients. Overall, the two populations displayed similar aberration profiles for the genes listed in Table 5. However, some differences were noted for the following genes; *THRB, RAF1, LPL, DCC, XIST, PCNT, STS,* as well as many genes on the 20q12-q13 cytoband (Table 5, Figure 1).

**Figure 2:**
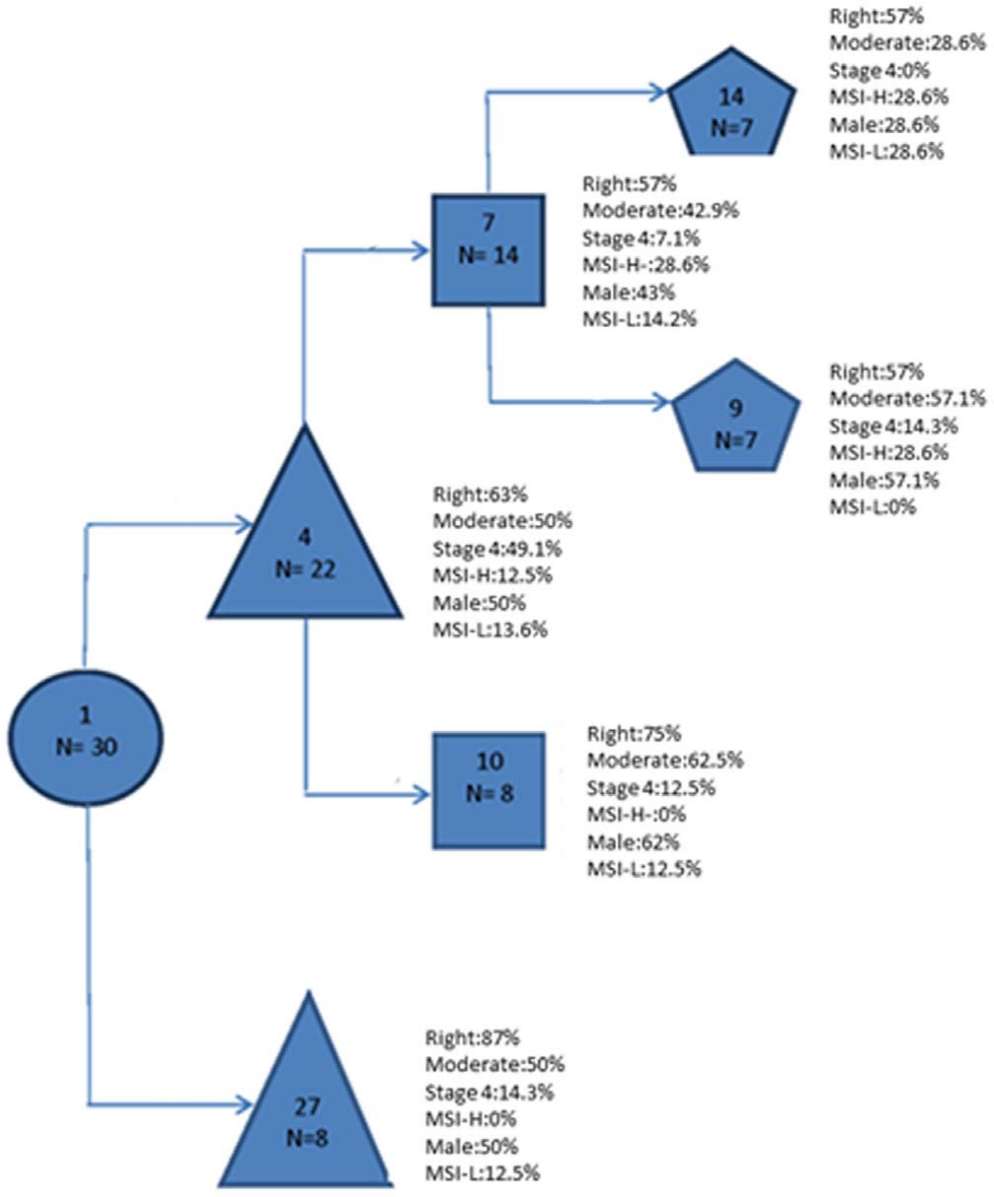
A schematic cladogram from a parsimony phylogenetic analysis of the aCGH data from the 30 CRC tumors. NC: No Changes, C: Changes, N: number of samples in cluster-The other digit within the clusters correspond to node numbers.

**Table 6:** Comparison of AA data with CAN genes' list from Sjoblom et al.

		African Americans (n = 30)	
Gene	Chromosome	Amplified (%)	Deleted (%)
*ABCA1*	9q31.1	1(3)	1(3)
*ACSL5*	10q25	1(3)	5(16)
*ADAM29*	4q34	0(0)	8(26)
*ADAMTS15*	11q25	3(10)	3(10)
*ADAMTS18*	16q23	11(36)	1(3)
*ADAMTSL3*	15q25.2	1(3)	6(20)
*APC*	5q22	2(6)	8(26)
*C10orf137*	10q26.1	1(3)	4(13)
*C15orf2*	15q11	0(0)	7(23)
*CD109*	6q13	8(26)	1(3)
*CD248*	11q13	11(36)	3(10)
*CD46(MCP)*	1q32	4(13)	3(10)
*CHL1*	3p26.1	2(6)	1(3)
*CNTN4*	3p26	4(13)	1(3)
*CSMD3*	8q23.3	14(46)	2(6)
*EPHA3*	3p11.2	3(10)	1(3)
*EPHB6*	7q34	18(60)	0(0)
*ERCC6*	10q11.2	3(10)	2(6)
*ERGIC3(SBDCAG84)*	20q12	20(66)	0(0)
*EVL*	14q32.2	4(13)	5(16)
*EXOC4(SEC8L1)*	7q31	18(60)	0(0)
*EYA4*	6q23	4(13)	2(6)
*FBXW7*	4q31.3	0(0)	8(26)
*GALNS*	16q24.3	14(46)	1(3)
*GNAS*	20q13.3	19(63)	0(0)
*GUCY1A2*	11q22	3(10)	6(20)
*HAPLN1*	5q14.3	1(3)	9(30)
*HIST1H1B*	6p22	11(36)	0(0)
*KCNQ5*	6q14	9(30)	1(3)
*KIAA1409*	14q32.1	5(16)	2(6)
*KRAS*	12p12.1	8(26)	1(3)
*KRT73(K6IRS3)*	12q13.3	11(36)	0(0)
*LGR6*	1q32.1	4(13)	3(10)
*LMO7*	13q22.2	17(56)	1(3)
*LRP2*	2q31	6(20)	2(6)
*MAP2*	2q34-35	6(20)	2(6)
*ACTL9*	19p13.2		
*MKRN3*	15q11	0(0)	5(16)
*MLL3*	7q36.1	16(53)	0(0)
*MMP2*	16q12-13	15(50)	1(3)
*NF1*	17q11.2	12 (40)	8(26)
*OBSCN*	1q42.1	4(13)	3(10)
*P2RX7*	12q24	8(26)	1(3)
*P2RY14*	3q25	6(20)	1(3)
*PHIP*	6q14	8(26)	1(3)
*PKHD1*	6p12.2	5(16)	1(3)
*PKNOX1*	21q22.3	7(23)	7(23)
*PRKD1*	14q11	2(6)	6(20)
*PTPRD*	9p23-24	1(3)	1(3)
*PTPRU*	1p35	6(20)	6(20)
*RET*	10q11.2	0	2(6)
*RUNX1T1*	8q22	14 (46)	2(6)
*SCN3B*	11q23.3	3(10)	3(10)
*SFRS6*	20q13.1	19(63)	0
*SLC29A1*	6p21	11(36)	2(6)
*SLC44A4(C6orf29)*	6p21.3	11(36)	2(6)
*SMAD2*	18q21.1	0	16 (53)
*SMAD3*	15q22.3	1(3)	6(20)
*SMAD4*	18q21.1	0	16 (53)
*SYNE1*	6q25	4(13)	2(6)
*TBX22*	Xq21.1	12 (40)	4(13)
*TCF7L2*	10q25.3	1(3)	5(16)
*TGFBR2*	3p22	4(13)	1(3)
*TP53*	17p13.1	10 (33)	10 (33)
*TTLL3*	3p25.3	5(16)	2(6)
*UHRF2*	9p24.1	1(3)	1(3)
*UQCRC2*	16p12	10 (33)	1(3)
*ZNF442*	19p13.2	12 (40)	7(23)

### Phylogenetic analysis of the CRC aCGH data

A maximum parsimony phylogenetic analysis was conducted on the CGH data through MIX algorithm (of the PHYLIP analytical package [25]) to produce the phylogenetic cladogram. The generated cladogram branched into two main clades and the partition and further subdivisions into clusters are summarized schematically in Figure 2. One clade included 22 patients (right-sided CRC: 63%; male: 50%; higher stage [>2]: 59%) that included all MSI-H samples. The other clade included 8 patients (right-sided CRC: 87%; male: 50%; higher stage >2: 71%, all were non-MSI. The first clade of 22 patients was further divided into two smaller groups with 14 (right sided CRC: 57%; male: 43%; higher stage [>2]: 43%) and 8 (right sided CRC: 75%; male: 62%; higher stage [>2]: 87%, p = 0.04) patients. It is noteworthy that 80% (4/5) of MSI-H tumors grouped together within the 14 patients' clade (Figure 2). All of these tumors have a very low number of aberrations (<15). The only MSI-H tumor that did not cluster with the others have a high number of aberrations (63) and as such, was most likely driven by chromosomal instability rather than by microsatellite instability.

## Discussion

Genome-wide studies have the potential to reveal genetic markers that may help explain the higher incidence of colorectal cancer in the African American population. We have previously conducted several studies on the role of MSI, methylation of CAN genes and mutations of known genes such as BRAF and KRAS (21–23, 25), as well as an aCGH analysis on a smaller number of CRCs from AA population (19). These studies were instrumental in revealing some of the specific genetic and epigenetic alterations that occur in this population. Herein, we elaborated upon our previous work and conducted a microsatellite instability analysis as well as whole genome analysis of copy number aberrations in CRC from AA patients (n = 30) with the goal of finding overlapping alterations between these two types of DNA variations. More specifically, phylogenetic clustering of the tumors based on copy number data was used to demonstrate that MSI-H tumors cluster together in the background of widespread chromosomal instability, a paradigm that has been poorly understood in the past.

The AA population analyzed in this study was relatively younger (mean age of 63.5 years) reflecting the disproportionate burden of CRC among African Americans [26], [27]. Seventy percent of the tumors were proximal confirming an observed population-based trend of tumor location in this population. While 90% of the tumors were moderately differentiated, more than 60% were higher than stage 2. These data suggest that many of these tumors would have dedifferentiated in a short amount of time leading to their invasiveness and metastasis. These data taken together shed some insight into the higher incidence and aggressiveness of CRC in AAs from clinical and pathological standpoints.

The MSI analysis revealed that 5 out of 30 tested tumors were MSI-H (17%). This MSI-H rate remains higher than that reported in the general population [24]. It is also noteworthy that 4 tumors were MSI-L.

There was an average of 25.46 copy number aberrations detected per tumor based upon our aCGH results. The MSI-H tumors alone showed a lower rate of 19.0 aberrations, while the non-MSI-H tumors showed 26.7 per tumor. This quantitative difference is supported by the concept that MSI-L tumors, unlike the MSI-H ones, are generally driven by chromosomal instability [28]. The overall number of aberrations did not seem to be associated with any of the clinico-pathological parameters (Table 4). There were tumors with a higher number of aberrations that seem to have the CIN phenotype, while there were others with fewer aberrations that are most likely an accidental-manifestation of MSI or have the CIMP phenotype. Our CGH analysis of a few colonic adenomas revealed more stable karyotypes with fewer aberrations (data not shown).

Chromosomes 3, 5, 7 and 8 were the most frequently altered in our group of patients. There are several publications reporting that these chromosomes contain cancer genes that are relevant to colon cancer, as well as in other cancers. Chromosome 3 contains *MLH1,* a DNA mismatch repair gene that leads to the MSI-H phenotype upon deletion, mutation or its transcription silencing [29]. *PPM1L,* another CRC gene on chromosome 3, was shown to have variable copy number in APC-negative familial adenomatous polyposis CRC [30]. Chromosome 5 displayed 41 aberrations equally distributed among deletions and amplifications [20/21]. *APC* is an important CRC gene on chromosome 5; *APC* plays a major role in the early steps on CRC events both in sporadic CRC as well as in hereditary FAP syndrome [31]. Chromosome 7 contains caretaker genes, such as *PMS2* [32], a DNA mismatch repair gene, and TSGs, such as *PIK3CG* [33]. Since TSGs are expected to be deleted, the disproportionate amount of gains [35 amplifications/12 deletions] on chromosome 7 quite intriguing. Chromosome 8 was the one with most aberrations [25 amplifications/23 deletions]. This chromosome is known as the hotspot for CRC tumor progression [34].

Another chromosome with an interesting pattern of aberrations was chromosome X, with 24 aberrations (14 amplifications/20 deletions). This chromosome has been described as the carrier of TSGs. Our previous findings, findings lend further support to our current results that chromosome X was preferentially amplified in male CRC patients [20]. Indeed, 10 out of 15 male patients displayed amplification for chromosome X in comparison to only 4 female patients. A similar finding was observed in Japanese male CRC patients [35]. This amplification might suggest that females with X chromosome allelic imbalance might be more prone to developing cancers.

A comparison of our data with those obtained in Caucasians [19] for 41 known oncogenes and TSGs revealed overall a similar aberration profile in the two populations. One interesting gene showing population-specific patterns is *Xist*, an RNA gene X whose expression determines the pattern of chromosome X inactivation in females [36]. *Xist* was amplified in approximately one third of both Caucasian and AA tumors, but was deleted only in AAs (13%). Other X- chromosome-related genes with differences between the populations were *STS* (steroid sulfatase) that was primarily deleted in Caucasians and amplified in AAs and *KAL1,* with a pattern similar to *Xist*. *STS* is known to be involved in female cancers, such as ovarian and breast cancers [37], [38], but not much is known about its potential role in CRC. *KAL1* was amplified and deleted in different subsets of our AA patients. Jian et al. have shown that *KAL1* gene expression is decreased in early stage and increased in later stages of cancers [39]. Their screening of colon, lung and ovarian cancer cDNA panels indicated significant decrease in *KAL1* expression in comparison to matching noncancerous tissues. This expression increased with the progression of cancer from earlier (I and II) to later (III and IV) stages of the cancer. These findings might reflect that the chromosomal aberrations observed in our set of samples are stage-specific. Among autosomal genes, *DCC* (Deleted in Colon Cancer) was deleted in 50% of the cases, unlike in Caucasians where it was more frequently amplified than deleted. Its status in AAs is more in line with its known function as a TSG and loss during colon oncogenic transformation [40]. Two contiguous genes on chromosome 3, *THRB* and *RAF1* are primarily amplified in AAs while the same genes were deleted in Caucasians. *THRB* gene was shown to act as an oncogene in thyroid carcinomas [41], but not much is known about its possible role in colon cancer. *RAF1* is known to be involved in many cancers (melanoma, gastric and prostate) through gene rearrangements along with other genes of the RAF family [42]. Three genes on chromosome 20 (*TPD521.2, TOP1* and *TNFRSF6B*) showed a much higher frequency of amplification in AAs than in Caucasians. Not much is known about *TPD521.2*. *TOP1* higher expression was shown to be associated with breast cancer, where it is a predictor of poor prognosis [43], but its role in colon cancer has not been established Antibody neutralization of *TNFRSF6B* in hepatocellular carcinoma cell lines inhibited proliferation and induced apoptosis [44]. This finding agrees with the higher amplification frequency of this gene in our cohort.

When we compared our data to another gene list established by Sjöblom et al. [21], we discovered that most of these genes were also altered in our population (Table 6), with 10 gene being predominantly deleted and 19 preferentially amplified. *TP53* was equally amplified and deleted in our set of samples (in 10 out of 30). It is well known that p53 (TP53) is a tumor suppressor gene [45] which fits more for its deletion profile rather than its amplification. *SMAD2* and *SMAD4* were the most frequently deleted genes in this cohort (in 16 out of 30). We have previously reported a different result in our aCGH analysis of 15 AA colon tumors [20]. However with more samples (n = 30) and improved analysis software (Genomic Workbench 6.5), our present findings are more in line with the known TSG status in many cancers [46]. Neurofibromin (NF1) that is also lost in many samples of our cohort is known to act a TSG in colon by turning the active form of Ras into an inactive form [47]. FBXW7, a component of the SCF (Skp1/Cullin/F-box protein) E3 ubiquitin ligase complex, acts as a tumor suppressor in several tissues and targets multiple transcriptional activators and proto-oncogenes for ubiquitin-mediated degradation. The gene *FBXW7*, which is deleted in many of our samples, influences murine intestinal homeostasis and cancer, targeting Notch, Jun, and DEK for degradation [48].

Regarding the amplified genes, CD248 (TEM-1) amplification in 11 samples might be justified by its established role in tumor angiogenesis [49]. While *EPHB6* is amplified in our cohort, its function is known to be a metastasis suppressor in non-small cell lung cancer [50], suggesting that it has a different function in colon tissue that needs to be characterized further. Another surprising discrepancy is that *MMP2* was amplified in our AA CRC tissues, while the use of MMP1/2 inhibitors has been shown to promote cell invasion of CRC cell lines in vitro [51]. *GNAS* was shown to be activated through amplification primarily in ovarian cancer [52] as well as through activating mutations in colorectal cancer [53]. Our data here confirm that GNAS activation through amplification occurs in colon as well. *GNAS* was shown to act through the activation of Wnt and ERK1/2 MAPK pathways as was shown in Apc(Min/+) mice [53]. *LMO7*, also amplified in our samples, was shown to mediate cell-specific activation of Rho-MRTF_SRF pathway, where it plays an important role in breast cancer cells migration [54]. While the above discussion centered on genes already known to be oncogenes or TSGs based on prior studies, most of the aberrations that were recorded by CGH affect genes without an annotated role in cancer. We used the occurrence of these unannotated aberrations in a phylogenetic clustering analysis of the tumors based on all recorded aberrations to see how these tumors might relate to one another and whether any clinical, pathological or molecular parameter might drive the nature of the chromosomal aberrations within a given tumor. These analyses revealed that gender, age and tumor location do not have an impact on the nature of the chromosomal aberrations. The expectation would be that poorly differentiated tumors would have had more time to accumulate more chromosomal aberrations in chromosomes of genes involved in cell differentiation characteristics. The most striking finding from our parsimony analysis was the clustering of 80% MSI-H tumors in the generated cladogram, separated from MSI-L and MSS tumors. The only MSI-H CRC that was an exception to this rule had a higher number of aberrations (63 aberrations) and as such should not be primarily defined as an MSI = H tumor but rather as a CIN tumor. The other MSI-H tumors had fewer aberrations (less than 15) than MSI-L and MSS tumors. The parsimony phylogeneticy analysis implies that this difference is not only quantitative, but also qualitative. This is in agreements with the results of Trautmann et al. (2006) regarding the difference in number and nature of chromosomal alterations between MSI and MSS tumors [18]. CGH array data are very informative. However, because many of the chromosomal aberrations span large genomic areas and affect many genes at once, it is difficult at the present time to assign weight and value to genes within a given aberration. To distinguish between driver genes and passenger genes within an aberration, one would need to complement CGH experiments with expression analysis to establish markers' whose differential expression associates with the oncogenic transformation. Such an integrative approach along with the inclusion of samples from non AA patients would allow the distinction between driver and passenger genes and would allow the identification of race specific aberrations, if any, in the colon oncogenic transformation [55].
